# The Efficacy and Safety of Sertaconazole Cream (2 %) in Diaper Dermatitis Candidiasis

**DOI:** 10.1007/s11046-013-9642-3

**Published:** 2013-04-02

**Authors:** Alexandro Bonifaz, Andrés Tirado-Sánchez, María José Graniel, Carlos Mena, Adriana Valencia, Rosa María Ponce-Olivera

**Affiliations:** 1 Department of Mycology, Hospital General de México OD, Dr. Balmis 148, Col Doctores, CP 06720 Mexico, DF Mexico; 2 Dermatology Service, Hospital General de México OD, Dr. Balmis 148, Col Doctores, CP 06720 Mexico, DF Mexico; 3 Dermatology Service, Hospital Infantil de Mexico, Mexico, DF Mexico

**Keywords:** Candidiasis, *Candida*, *Candida albicans*, Diaper dermatitis, Imidazole, Sertaconazole

## Abstract

**Aim:**

Diaper dermatitis (DD) is an inflammatory irritating condition that is common in infants. Most cases are associated with the yeast colonization of *Candida* or diaper dermatitis candidiasis (DDC), and therefore, the signs and symptoms improve with antimycotic treatment. Sertaconazole is a broad-spectrum third-generation imidazole derivative that is effective and safe for the treatment for superficial mycoses, such as tineas, candidiasis, and pityriasis versicolor. Our goal was to assess the efficacy and safety of sertaconazole cream (2 %) in DDC.

**Materials and methods:**

Twenty-seven patients with clinical and mycological diagnosis of DDC were enrolled and treated with 2 daily applications for 14 days and were followed-up for 2 further weeks.

**Results:**

Three etiologic agents were isolated: *Candida albicans* in 88.8 %, *Candida parapsilosis* in 7.3 %, and *Candida glabrata* in 3.2 %. There was an average symptom reduction from 7.1 to 3.2 in the middle of treatment and to 1.2 and 0.4 units at the end of treatment and follow-up, respectively. The treatment evaluation at the end of the follow-up period showed a total clinical and mycological cure in 88.8 %, improvement in 3.7 %, and failure in 7.4 %. There was side effect (3.7 %) of skin irritation, but the drug was not discontinued.

**Conclusions:**

Based on its safety and effectiveness, sertaconazole cream may be considered a new alternative for DDC treatment.

## Introduction

Diaper dermatitis (DD) is a highly frequent condition in infants, particularly at 1–15 months of age. Its onset occurred in the 1960s with the development of disposable diapers. It is an irritating and inflammatory acute dermatitis in the perineal and perianal areas resulting from the occlusion and irritation caused by diapers. DD is directly influenced by a series of factors, such as excessive humidity and skin maceration, which regularly tends to show a change in pH, thereby making it more alkaline. This is due to urea transformation into ammonium hydroxide, which favors the loss of the skin barrier and subsequent colonization by various microorganisms [1–4].

Colonization by different *Candida* species, predominantly *Candida albicans,* occurs in 70–80 % of DDs, and therefore, it may be considered diaper dermatitis candidiasis (DDC), which is also called diaper rash *Candida*. Clinically, this condition occurs in the region covered by the diaper, affecting the gluteal area, perineum, groin, and, occasionally, part of the genitalia. In terms of morphology, it shows erythematous, scaly, macerated plaques with edema, occasionally accompanied by vesicles and pustules. It has symptoms of burning and itching, but these data are hard to evaluate because they occur in young children [5]. It should be noted that DD is an inflammatory and irritating acute condition that is not included within mycoses; its association with different yeasts derives from its colonization [6, 7]. In general, DD may be treated with desiccants and inert substances. However, when the presence of *Candida* yeasts is confirmed, it is necessary to provide antimycotic treatment, which regularly tends to improve signs and symptoms, together with an explanation that diapers should be changed more frequently, desiccants should be used, and humidity and maceration should be avoided. [4, 7, 8].

Sertaconazole nitrate is a broad-spectrum third-generation benzothiophene imidazole against dermatophytes, *Candida*-type yeasts, filamentous fungi, and some bacteria [9, 10]. It is used for the treatment for various tineas, candidiasis (vaginal), and pityriasis versicolor. Its antimycotic action is based on its fungistatic and fungicidal action, with long skin perdurability for up to 72 h. Its mechanism of action is based on inhibiting cytochrome p-450, on which ergosterol synthesis is dependent. Ergosterol is the main component of the fungal membrane. Its pharmacodynamics has been described with good tolerability, few side effects, and high efficacy against major superficial mycoses [10, 11].

Based on the properties of sertaconazole nitrate 2 %, we decided to conduct an efficacy and safety study in patients with *Candida* colonization in DD.

## Materials and Methods

A descriptive, prospective, open-label, and non-comparative study to evaluate the efficacy and safety of sertaconazole cream 2 % in patients with DDC was conducted. Children from the dermatology units of the Hospital General de México OD (Mexico General Hospital) and the Hospital Infantil de México (Mexico Children’s Hospital) were enrolled. Parents or legal guardians accepted to take part in the study in writing, and the protocol was followed based on the Declaration of Helsinki and its subsequent amendments.

**Fig. 1:**
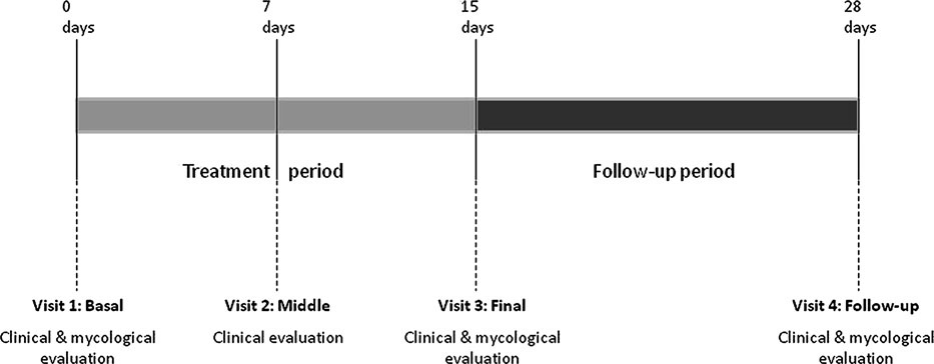
Study treatment and follow-up scheme

The study design included four mandatory visits: baseline Visit 1 at the beginning of the study; intermediate Visit 2, after 7 days of medication; final Visit 3 after 14 days of medication and evaluation one day later without medication (Day 15); and follow-up Visit 4 after 14 days without medication. Clinical evaluation was performed in all 4 visits, and mycological evaluation took place in Visits 1, 3, and 4. The study scheme is shown in Fig. 1.

Inclusion criteria: male or female children, between 2 and 24 months of DDC. Only patients who had the condition for less than 4 months were included. Four signs were evaluated clinically: erythema, scaling, maceration, and edema. Each of these signs was scored 0–3, with the following codes: 0 = normal or without clinical data; 1 = mild; 2 = moderate; and 3 = severe. The following mycological studies were conducted: Direct examination with KOH 10 % to show mycological elements it is meaning the presence of pseudohyphae and/or blastoconidia; isolation of *Candida* strains in Sabouraud dextrose agar and CHROMagar-Candida^®^ media. Once obtained, the strains were identified by the commercial API-yeast 20^®^ system.

Exclusion criteria: patients who received topical antifungal within 2 months; patients with DD for more than 4 months; patients with associated conditions in the same region, such as associated bacterial infection (*Streptococcus* sp., *Staphylococcus* sp.), psoriasis, contact dermatitis, immunosuppressed patients, and patients on another type of topical medication in the diaper area.

The mother, or person in charge of each child, was instructed to apply sertaconazole cream 2 %. Two applications, one in the morning and one at night, were performed after washing the child and changing the diaper. Each mother was given 2 tubes of sertaconazole cream 2 % on Visits 1 and 2. No other drug was applied throughout the study, and the children were washed with soap and water only. After Visit 3, follow-up was made for 14 days without medication; the tubes provided were collected on Visits 2 and 3.

At the end of the study, a clinical evaluation was performed based on the measurement parameters; cure was considered: no clinical data and negative culture; improvement: discrete clinical data and negative culture; and failure: evident clinical data and positive culture.

Statistical analysis: Sample size was determined by recruitment time (4 months). The data obtained are presented in percentage values and bar graphs.

## Results

Twenty-seven—15 females and 12 males—cases of DDC with clinical and mycological diagnosis that complied with the inclusion criteria were enrolled. The youngest patient was 2 months old and the oldest was 22 months, with an average of 5.7 months. The main demographic, clinical, and mycological data are shown in Table 1; results from the clinical, therapeutic, and mycological evaluations are shown in Table 2.

**Table 1:** Main demographic, clinical, and mycological data

Data	Results
Number of patients: 27	15 females (55 %) 12 males (45 %)
Age	Youngest: 2 months Oldest: 22 months Average: 5.7 months
Condition evolution	Shortest: 4 days Longest: 120 days Average: 14.5 days
Condition classification (based on the clinical signs score)	Mild: 15 cases (55 %) Moderate: 10 cases (37 %) Severe: 2 cases (8 %)
Direct examination	Pseudohyphae + blastoconidia: 26/27 cases (96.3 %) Blastoconidia (plenty)^a^ 1/27 cases (3.7 %)
Cultures	*Candida albicans*: 24/27 (88.8 %) *Candida parapsilosis*: 2/27 (7.4 %) *Candida glabrata*: 1/27 (3.2 %)^a^

^a^The image of plenty blastoconidia only corresponded to the *C. glabrata* isolate

**Table 2:** Results of the clinical, mycological, and therapeutic assessments at all four visits

	Visit 1 (baseline) Day 0	Visit 2 (intermediate) Day 7	Visit 3 (final) Day 15	Visit 4 (follow-up) Day 28
Average of clinical signs^a^	7.1	3.2	1.2	0.4
Direct examination	Ps + B: 26 cases. B: 1 case	NP	Ps + B: 1 case	Ps + B: 2 cases
Cultures	*C. albicans:* 24 *C. parapsilosis:* 2 *C. glabrata:* 1	NP	*C. albicans*: 1 (3.7 %)	*C. albicans*: 2 (7.4 %)
Treatment evaluation	NP	NP	Cure: 24/27 (88.8 %) Improvement: 2/27 (7.4 %) Failure: 1/27 (3.7 %)	Cure: 24/27 (88.8 %) Improvement: 1/27 (3.7 %) Failure: 2/27 (7.4 %)
Side effects	–	–	1 case (3.7 %) with increased erythema	No erythema

*NP* not performed, *Ps* Pseudohyphae, *B* Blastoconidia

^a^Clinical signs (erythema, scaling, maceration, and edema), minimum average = 0, maximum average = 9

## Discussion

Diaper dermatitis is one of the most common skin conditions in the first months of life; its onset is due to leaving diapers on for long periods or to sensitivity. Its pathogenesis has been discussed for many years. The most important roles are played by humidity and skin maceration, as well as alkalinity resulting from urine and feces [3, 12–16]. In most instances, correcting these factors is enough for the skin to keep its balance. However, when humidity and occlusion factors persist, the skin barrier breaks and facilitates microbial colonization, which may occur by different bacteria, especially *Staphylococcus aureus* [5, 7]. Its main complication is colonization by *Candida* yeasts; they come from the intestine and are usually part of skin’s normal flora, particularly near the genitalia [3, 6, 7].

For many authors, *Candida* yeasts are present only as colonization, not as etiologic agents, and they are usually present in 70–80 % of DD [5, 6]. Nevertheless, when these yeasts have a virulent form (pseudohyphae, hyphae and/or large clusters of blastoconidia), they stop being associated with the flora and start to be considered as a superficial candidiasis, affecting the stratum corneum. In these cases, the use of antifungals or antimycotics such as nystatin, azole derivatives (clotrimazole, ketoconazole, etc.), or ciclopirox significantly reduces the clinical signs and symptoms, but it should be emphasized that, if humidity, maceration, and occlusion factors persist, recurrence is common [17–19].

This is an open-label, non-comparative study to evaluate the effectiveness and safety of sertaconazole cream. This imidazole derivative was used because of its good effectiveness within in vitro studies and in treating superficial and vaginal candidiasis, and its broad-spectrum, perdurability, and good tolerance [9, 11].

A demographic analysis of the data in Table 1 shows that both genders are balanced, and the average age is 5.7 months. The literature indicates that DD occurs between 1 and 15 months [3, 4, 7], with a higher average between 2 and 9 months. It is important to note that the mean duration of disease was 14.5 days because the limit was 4 months in our inclusion criteria. This is because it is harder to correct factors and get a treatment response in chronic DD cases. Some studies, for example, Hoeger et al. [20] take a limit of 6 months. It should be highlighted that most included cases were in a mild (55 %) to moderate (37 %) range, which also plays a role in getting a faster response to treatment and correcting predisposing factors.

From a mycological standpoint, it should be said that all DD cases were associated with virulent forms of *Candida*, that is, there were pseudohyphae in 26/27 patients (96.3 %) and it was only in one case (1/27; 3.7 %) that there was a significant cluster of yeasts corresponding to *C. glabrata,* which is a species that does not form these structures and its pathogenicity is marked with the number of yeasts of blastoconidia. *C. albicans* was predominant (88.8 %), similar to reports in the literature, but it is noteworthy that two further species were obtained: *C. parapsilosis* and *C. glabrata*, both related to resistance to different antifungals [21, 22].

Clinical, mycological, and therapeutic data may be seen in Table 2. Given that most included cases were mild or moderate, the overall average of clinical signs (erythema, scaling, burning, and edema) was 7.1 units. This is considered the baseline value, and it is clearly seen that, at the 7-day assessment, it goes down to 3.2, that is, more than 50 % of signs are considerably decreased. By the end of treatment, the average is 1.2 units, and it is even lower during follow-up. This is probably due to the same correction of predisposing factors; this reduction in signs such as erythema and edema may be due to sertaconazole’s good antimycotic action, but also to its important proved anti-inflammatory action [23, 24]. From a mycological standpoint, only one case failed by the end of treatment and two did during follow-up. According to the cure criteria, at the end of follow-up, there was clinical and mycological cure in 24/27 cases (88.8 %), improvement (i.e., clinical data and no-yeast isolation) in 1/27 case (3.7 %), and failure (persistence of clinical data and new isolation of yeast) in 2/27 cases (7.4 %). Failures corresponded to *C. albicans* species; one case was considered severe, and the other was considered moderate in the baseline classification. There was only one adverse event (3.7 %) of dermatitis at the end of treatment, with slightly increased erythema. The mother considered it possibly due to the medication and did not discontinue treatment. In the follow-up visit, the child did not show any clinical data and was considered a complete cure. The event was reported as a possible side effect.

In other studies reported in the literature on the use of antifungals in DD, the best outcomes are reported with the use of topical antifungals for 2 weeks. Even though ours is not a comparative study, it is similar to the study by Hoeger et al. [20], in terms of design and clinical–microbiological evaluation. They reported clotrimazole superiority over nystatin with complete (clinical and mycological) cure rates of 68.1 and 49.8 %, respectively, with a statistically significant difference. This is a study that enrolled more than 200 infants and used miconazole 0.25 % [19] in ointment; good results were also obtained particularly versus placebo.

Gallup et al. [25] conducted an open-label, non-comparative study using ciclopirox suspension (0.77 %) applied twice daily, but only for 7 days, and assessed after 14 days. They obtained good results in total success scores (*p* < 0.047), with a significant reduction in signs, symptoms, and mycological cure evaluation.

It is important to highlight that, even though *Candida* DD is a very frequent entity, few papers dealing with treatment are found in the literature. However, the new breathable disposable diaper technology has proved to reduce *Candida* prevalence in 35–80 %. This is important because airing the diaper area undoubtedly reduces yeast development in a significant way [26].

In general, azole derivatives have a broad spectrum of action in infections caused by dermatophytes and yeasts. Sertaconazole nitrate is a benzothiophene imidazole, a topical broad-spectrum antifungal developed for the treatment for skin and mucosal infections [9–11]. It has a dual mechanism of action: firstly, through ergosterol synthesis inhibition by blocking the enzymatic pathway of cytochrome p-450, which acts with cell growth; secondly, because it binds directly to non-sterol lipids on the fungal membrane and interferes with ligands from the intra-cellular contents, thereby causing cell death. It is an effective fungicidal and fungistatic agent. In addition, anti-inflammatory properties have been described by reducing cytokine secretion from activated lymphocytes, which controls the inflammatory component of dermatophytosis and candidiasis [1, 11].

## Conclusions

Sertaconazole cream 2 % for the treatment for DD-associated candidiasis achieved a >50 % reduction in clinical signs after 7 days of treatment (from 7.1 to 3.2 units) and practically symptomless by the end of treatment (0.4 units). It was effective for a clinical–mycological cure in 88.8 %. There was a possible side effect (3.7 %) of skin irritation that did not call for drug discontinuation. Based on the above, we conclude that sertaconazole cream is a broad-spectrum third-generation imidazole with great skin penetration and perdurability that may be considered as an alternative for the treatment for DD-associated candidiasis due to its high efficacy and good safety.
